# HtrA2/Omi: potential therapeutic targets for neurodegenerative diseases

**DOI:** 10.3389/fphar.2026.1705666

**Published:** 2026-01-13

**Authors:** Liting Xu, Min Zeng

**Affiliations:** Department of Nephrology, People’s Hospital of Longhua, Shenzhen, China

**Keywords:** HtrA2, neurodegenerative diseases, mitochondria, apoptosis, UCF-101

## Abstract

High temperature requirement protein A2 (HtrA2/Omi), a key regulator of mitochondrial quality control, plays a pivotal role in determining cell fate through its subcellular localization, whether mitochondrial or cytosolic. Growing evidence links the absence or dysfunction of HtrA2 to the pathogenesis of neurodegenerative diseases. This review examines the structure and function of HtrA2, highlights its transcriptional regulators, explores its involvement in neurodegeneration, and outlines the currently identified agonists and inhibitors, offering new insights for developing HtrA2/Omi as a potential therapeutic target for neurodegenerative disorders.

## 1 Introduction

The High Temperature Requirement A (HtrA) family comprises highly conserved serine proteases (Qiu et al., 2025), initially identified as periplasmic proteins essential required for high-temperature tolerance in *Escherichia coli*. These proteins function both as serine proteases and chaperones, with their activity regulated by temperature (Bhuiyan and Fukunaga, 2008). Low temperatures promote their chaperone function, while high temperatures activate their protease activity (Bhuiyan and Fukunaga, 2008). In humans, four isoforms of HtrA have been identified (HtrA1-HtrA4), with HtrA2 being the most extensively studied (Rosochowicz et al., 2024). HtrA2, also known as Omi, a stress-regulated endoprotease or serine protease (Faccio et al., 2000), is primarily located in the inner mitochondrial membrane under normal conditions. However, it translocates to the cytoplasm in response to hypoxia or heat stress, where it plays a pivotal role in regulating apoptosis and autophagy (Goo et al., 2017). As a key regulator of mitochondrial and cellular homeostasis, HtrA2 has attracted significant attention in the context of neurodegenerative diseases. Functional abnormalities of HtrA2 are closely associated with conditions such as Parkinson’s disease (PD) and Alzheimer’s disease (AD) (Goo et al., 2017). This article reviews the biological functions of HtrA2, summarizes recent research on its role in neurodegenerative diseases, and explores its potential as a therapeutic target for these disorders.

## 2 Structural characteristics of HtrA2

Currently, four subtypes of HtrA have been identified in humans. All share a conserved protease domain and a C-terminal PDZ domain, yet exhibit significant differences in their N-terminal regions. The N-terminal regions of HtrA1, HtrA3, and HtrA4 contain a signal peptide (SP), an insulin-like growth factor binding protein domain (IGFBP), and a Kazal-type serine protease inhibitor domain (Kaz), while HtrA2’s N-terminal region consists of a transient peptide (TP) and a transmembrane domain (TM) (Liu et al., 2024). These structural differences contribute to their functional divergence, with the most extensive research conducted on HtrA2. The full-length HtrA2 protein comprises 458 amino acids, and it is the only mitochondrial protease with a PDZ domain (amino acids 365–455), which facilitates recognition of exposed hydrophobic regions on misfolded proteins, enabling efficient degradation (Clausen et al., 2011; Singh et al., 2011). Additionally, HtrA2 undergoes cleavage at its 133 N-terminal residues to form the activated version, which translocates from the mitochondria to the cytoplasm under stress. This cleavage exposes a tetrapeptide motif (Ala-Val-Pro-Ser, AVPS) that binds to the inhibitor of apoptosis proteins (IAPs). AVPS specifically interacts with the second baculoviral IAP repeat (BIR2) domain of X-linked inhibitor of the apoptosis protein (XIAP), counteracting its inhibition of apoptosis and thereby promoting cell death. Furthermore, the first 60 amino acids of HtrA2 serve as a mitochondrial targeting sequence (MTS), allowing its import into mitochondria to carry out its functions (Su et al., 2019). Given HtrA2’s pivotal role in regulating cell survival, identifying its regulatory factors is critical for understanding its broader implications in cellular processes.

## 3 Regulation of HtrA2

### 3.1 p53

As a tumor suppressor gene, p53 functions as a negative regulator of the cell cycle. Approximately 50% of human cancers are linked to genetic mutations that result in the loss of p53 function (Liu et al., 2025; Toledo, 2025). Upon activation, p53 binds to the promoters of target genes, initiating the expression of multiple genes involved in regulating critical biological processes such as the cell cycle, DNA repair, differentiation, and apoptosis (Dyson and Wright, 2025; Koo et al., 2025). Notably, p53 has been implicated in the transcriptional regulation of HtrA2. Jin et al. demonstrated that p53 induces apoptosis through serine protease activity in *Drosophila*, where the protease-mediated inactivation of CIAP1 occurs in a p53-dependent manner. Inhibition of CIAP1 cleavage by the serine protease inhibitor AEBSF completely blocked p53-dependent apoptosis (Jin et al., 2003). Mechanistically, p53 activation promotes the transcription of HtrA2, facilitating its interaction with and cleavage of CIAP1, which releases caspase inhibition and activates apoptosis (Jin et al., 2003). Additionally, through bioinformatics analysis, luciferase reporter assays, and chromatin immunoprecipitation (CHIP) confirmed that p53 specifically binds to the HtrA2 promoter to enhance its transcriptional expression (Liu et al., 2016). In aged myocardium, increased p53 expression and its binding to the HtrA2 promoter were observed, leading to elevated HtrA2 protein levels (Wu et al., 2019). Similarly, adenoviral expressing of p53 increased HtrA2 mRNA levels and induced neuronal apoptosis (Tun et al., 2007). These findings highlight the potential of targeting p53 to regulate HtrA2 expression as an effective strategy to control apoptosis.

### 3.2 Heat shock factor 1 (HSF1)

HSF1, a key regulator of the heat shock response, was discovered in 1984. HSF1 is activated by various cellular stresses, such as heat shock, and induces the expression of heat shock proteins to protect the proteome and help cells survive these acute stresses (Fiser and Muller, 2025). As a helix-turn-helix transcription factor, HSF1 regulates gene expression by binding to heat shock elements in the promoters of target genes (Amin et al., 1988; Xiao and Lis, 1988). Liu et al. predicted, through bioinformatics analysis, that there are eight HSF1 binding sites in the HtrA2 promoter, and ChIP analysis confirmed that HSF1 binds to this promoter (Liu et al., 2016). In senescent cardiomyocytes, upregulation of HSF1 expression promotes Omi/HtrA2 expression by enhancing its promoter activity, ultimately accelerating the apoptosis of these cells (Liu et al., 2019).

In addition to transcriptional regulation, HtrA2 expression and activity are modulated by various other factors. Its homotrimeric structure and C-terminal PDZ domain are essential for its enzymatic activity. Molecules such as reactive oxygen species (ROS), presenilin 1, and PINK1 have been shown to enhance the hydrolytic enzyme activity of HtrA2 (Su et al., 2019). Point mutations, including those at serine residues S400 and S142, also affect HtrA2’s proteolytic function (Su et al., 2019). Furthermore, certain compounds can regulate HtrA2 expression and activity, which will be discussed in subsequent sections.

## 4 The function of HtrA2

**FIGURE 1:**
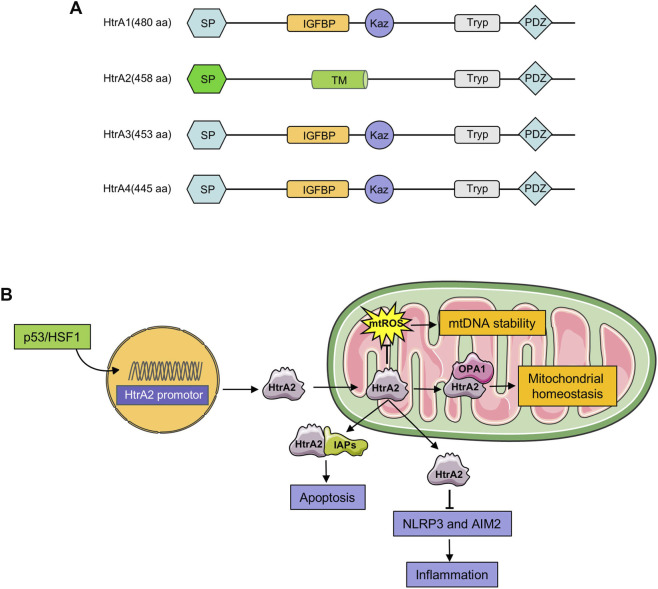
Structural domains of the HtrA protein and biological functions of HtrA2. **(A)** The structural domains of the four subtypes of HtrA protein subtypes. **(B)** The expression of HtrA2 is transcriptionally regulated by the transcription factors p53 and HSF1. Following expression, HtrA2 translocates to the mitochondria, where it exerts an antioxidant stress effect, preserving mtDNA stability of and mitochondrial homeostasis. When HtrA2 is released from the mitochondria into the cytoplasm, it interacts with IAPs to promote apoptosis.

As a double-edged sword within cells, HtrA2 is essential for maintaining cellular homeostasis. Current research on HtrA2 primarily focuses on its roles in apoptosis, mitochondrial homeostasis, and inflammation (Figure 1). This review summarizes the latest findings regarding HtrA2’s involvement in these processes.

### 4.1 Regulation of apoptosis

Mitochondria-mediated apoptosis is a critical pathway of programmed cell death. In response to various stressors, including calcium overload, oxidative stress, DNA damage, and growth factor deprivation, cytochrome c is released from the mitochondrial intermembrane space into the cytoplasm (Vogler et al., 2025; Hall-Younger and Tait, 2025). In the presence of ATP/dATP, cytochrome c triggers the assembly of the apoptosome, a protein complex that includes apoptotic protease activating factor-1 (Apaf-1) and pro-caspase-9 (Xu et al., 2021; Shakeri et al., 2017; Sever et al., 2023; Morse et al., 2024). This assembly activates pro-caspase-9, which subsequently activates pro-caspase-3 and pro-caspase-7, ultimately leading to apoptosis (Brentnall et al., 2013; Nandi et al., 2024). Various families of IAPs, such as cellular IAPs (cIAPs), melanoma IAP (ML-IAP), and XIAP function primarily to block apoptosis by inhibiting caspases 3, 7, and 9 (Michie et al., 2020; Fan et al., 2025). HtrA2, the first identified IAP-binding protein, acts as a negative regulator of IAPs (Jin et al., 2003). In response to apoptotic signals, HtrA2 is cleaved and activated within mitochondria and subsequently released into the cytoplasm. Once in the cytoplasm, the AVPS domain of HtrA2 is exposed, allowing it to bind to IAP domains, catalyzing the inactivation of IAPs, deregulating caspase activity, and initiating caspase-dependent apoptosis (Srinivasula et al., 2003; Yang et al., 2003). Furthermore, due to its serine protease activity, HtrA2 can cleave cytoskeletal proteins, including actin, α-/β-tubulin, and vimentin, disrupting the cytoskeletal structure and inducing caspase-independent apoptosis (Vande et al., 2007). Increasing evidence highlights the pivotal role of HtrA2 in apoptosis, and targeting HtrA2 to inhibit apoptosis presents a potential therapeutic strategy for various of diseases, particularly cancer.

### 4.2 Mitochondrial homeostasis

Under normal conditions, HtrA2 is a serine protease that located in the mitochondrial intermembrane space, playing a key role in maintaining mitochondrial homeostasis. Knockdown of HtrA2 leads to an accumulation of ROS, reduced mitochondrial membrane potential, and impaired mitochondrial function (Kieper et al., 2010). HtrA2 also selectively recognizes and interacts with the NAC region of α-synuclein (α-Syn), promoting its hydrolysis, and preventing α-Syn accumulation within mitochondria, thereby inhibiting mitochondrial ROS production (Nam et al., 2023). In mouse brain tissue, loss of HtrA2 protease activity triggers ROS production, resulting in conformational changes in mitochondrial DNA (mtDNA), which cause mtDNA nicks and mutations. However, overexpression of HtrA2 with mitochondrial protease activity restores mtDNA conformational stability (Goo et al., 2013), highlighting the indispensability of HtrA2’s protease activity in maintaining mtDNA homeostasis. Additionally, HtrA2 deficiency impairs abnormal mitochondrial oxidative phosphorylation. Favreau et al. found that HtrA2 knockout cells exhibited increased proton translocation via ATP synthase, accompanied by reduced ATP production and truncation of the F1 α subunit (Plun-Favreau et al., 2012). HtrA2 is also involved in regulating of mitochondrial dynamics. Optic atrophy protein 1 (OPA1), a key regulator of mitochondrial dynamics, is affected by mutation and increased proteolysis, leading to mitochondrial dysfunction (Maremanda et al., 2021). In HtrA2 knockout mouse embryonic fibroblasts, elongated mitochondria and upregulation of soluble OPA1 were observed. However, overexpression of Omi/HtrA2, but not the protease mutant [S306A] HtrA2, in these cells restored mitochondrial morphology and OPA1 expression (Kieper et al., 2010). Furthermore, co-immunoprecipitation confirmed a direct interaction between HtrA2 and OPA1, regulating mitochondrial dynamics (Kieper et al., 2010). A similar effect was observed in mice with neural-specific deletion of HtrA2, where mitochondria in the cerebellum exhibited abnormal morphology, including swelling, blistering, and cristae fragmentation, accompanied by defective OPA1 processing by 20 days of age (Patterson et al., 2014). Severe defects in OPA1 processing were also detected in fibroblasts from patients with HtrA2 mutations (Olahova et al., 2017), and inhibition of HtrA2 by chemotherapeutic agents effectively reduced OPA1 expression (Huang et al., 2023). Considering the dual roles of HtrA2 in apoptosis and mitochondrial homeostasis, its function in cells can be seen as a double-edged sword. In the mitochondria, HtrA2 prevents the production of mtROS, maintains mitochondrial oxidative phosphorylation, and regulates mitochondrial dynamics. When released into the cytoplasm, however, it activates the apoptotic pathway.

### 4.3 Inflammation

Chronic aseptic inflammation is a pathological hallmark in the progression of a various diseases, and targeted anti-inflammatory therapy is a key treatment strategy for some conditions. Inflammation serves to eliminate the specific causes of cellular damage, with low-grade inflammation typically benefiting the disease outcome. However, when pathogenic factors persist, excessive inflammation can lead to further tissue damage, exacerbating disease progression (Sotak et al., 2025). Studies have increasingly highlighted the critical role of HtrA2 in regulating inflammation. Gervais et al. demonstrated that disrupting HtrA2 protease activity in macrophages, both *in vitro* and *in vivo*, led to the activation of the NLRP3 and AIM2 inflammasomes (Rodrigue-Gervais et al., 2018). Mechanistically, HtrA2 regulates autophagy through its protease activity, preventing the accumulation of the inflammasome adaptor apoptosis-associated speck-like protein containing a CARD (ASC), thus inhibiting inflammasome activation (Rodrigue-Gervais et al., 2018). On the other hand, HtrA2 also plays a role in promoting inflammatory responses, as shown in a collagen-induced arthritis model. Xu et al. found that HtrA2 deficiency reduced proinflammatory cytokine production in lipopolysaccharide (LPS)- or CpG-induced bone marrow-derived macrophages (BMDMs) (Xu et al., 2021). Further investigations revealed that HtrA2 interacts with TNF receptor-associated factor 2 (TRAF2) and stabilizes TRAF2, which is crucial for regulating inflammation in BMDMs (Xu et al., 2021). In patients with rheumatoid arthritis (RA), HtrA2 levels in synovial fluid were elevated compared to those in osteoarthritis (OA) patients, and the concentration of HtrA2 correlated with immune cell numbers in the synovial fluid of RA patients (Jeong et al., 2023). Moreover, HtrA2 levels were significantly associated with the severity of synovitis and the expression of proinflammatory cytokines and chemokines. Knockdown of HtrA2 reduced the release of these cytokines and chemokines (Jeong et al., 2023), suggesting that HtrA2 acts as an inflammatory mediator and may induce inflammation. Inhibiting its expression could therefore provide a potential approach to mitigate inflammation. HtrA2 also contributes to inflammatory diseases by regulating necroptosis. Proteomic analysis revealed downregulation of HtrA2 expression in dextran sulfate sodium (DSS)-induced colitis, accompanied by increased necroptosis in intestinal epithelial cells. Inhibition of HtrA2 (using the inhibitor UCF-101) or silencing HtrA2 expression reduced necroptosis (Zhang et al., 2019). In response to stimuli, HtrA2 translocates from the mitochondria to the cytoplasm, where it interacts with RIPK1, a key protein in necroptosis regulation (Zhang et al., 2019). These findings indicate that HtrA2 plays a variable role in inflammation regulation across different disease models and stages. A better understanding of HtrA2’s role in inflammation is crucial for its potential as a therapeutic target for inflammatory diseases.

## 5 HtrA2 in neurodegenerative diseases

**FIGURE 2:**
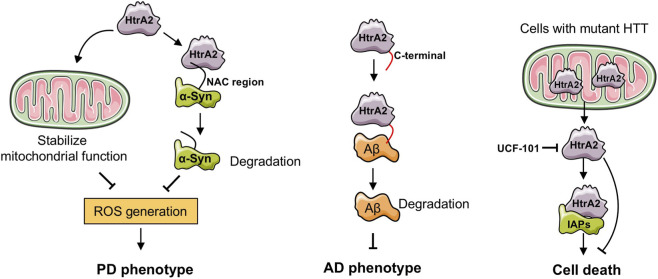
The role of HtrA2 in neurodegenerative diseases. Under normal conditions, HtrA2 degrades abnormal proteins (α-Syn or Aβ) and inhibits ROS, thus maintaining mitochondrial function and slowing the progression of AD and PD. In cells expressing the mutant Huntington protein, abnormal release of HtrA2 leads to a reduction in the levels of IAPs in cytoplasmic IAP levels, resulting in cell death. However, pharmacological inhibition of HtrA2 can promote cell survival.

Neurodegenerative diseases are prevalent among the elderly, and with the aging population, their incidence continues to rise. Numerous studies have highlighted that mitochondrial dysfunction and impaired dynamics contribute significantly to the development of neurodegenerative diseases (Antico et al., 2025; Mani et al., 2025). As a critical regulator of mitochondrial homeostasis, HtrA2 plays a pivotal role in the pathogenesis of these disorders. Increasing evidence links the absence or dysfunction of HtrA2 to various neurodegenerative diseases (Figure 2). This involvement extends beyond its role as an “apoptotic protease,” encompassing diverse processes such as mitochondrial homeostasis, autophagy, synaptic plasticity, and neuroinflammation.

### 5.1 Parkinson’s disease (PD)

The association between HtrA2 and PD was first identified through genetic mutations in HtrA2 in sporadic PD patients (Strauss et al., 2005; Bogaerts et al., 2008). The HTRA2 p.G399S mutation is linked to hereditary essential tremor, with homozygous carriers of this allele eventually developing PD (Unal et al., 2014). Immunohistochemical analysis has also shown strong HtrA2 expression in the brains of PD patients (Kawamoto et al., 2008), while HtrA2 knockout mice exhibit a Parkinsonian neurodegenerative phenotype (Martins et al., 2004; Jones et al., 2003), characterized by the accumulation of misfolded proteins in mitochondria (Moisoi et al., 2009). This suggests that abnormal protease activity of HtrA2, leading to the buildup of misfolded proteins in mitochondria, may play a role in the onset and progression of PD. The proteolytic activity of HtrA2 is tightly regulated to prevent undesired proteolysis. Under normal conditions, the interaction between the protease domain and the regulatory PDZ domain of HtrA2 inhibits its protease activity. This inhibition is reversed when the PDZ domain binds to a peptide from a misfolded protein (Ruiz et al., 2006; Clausen et al., 2011; Desideri and Martins, 2012). Furthermore, oxidative stress mediated by HtrA2 is another potential mechanism contributing to PD symptoms. Nam et al. demonstrated that HtrA2 can specifically interact with the NAC region of α-Syn, promoting its hydrolysis, and preventing its accumulation in mitochondria, thereby reducing mitochondrial ROS production. In contrast, HtrA2 knockout promotes the generation of mitochondrial ROS mediated by α-Syn (Nam et al., 2023). Similar findings showed that the absence of HtrA2 leads to mitochondrial respiratory dysfunction and increase ROS production (Moisoi et al., 2009). Moreover, treatment of HtrA2 knockout mice with Idebenone, a synthetic antioxidant from the coenzyme Q family, delayed the onset of PD-like symptoms and extended lifespan (Gerhardt et al., 2011).

### 5.2 Alzheimer’s disease (AD)

AD is a prevalent neurodegenerative disorder globally, characterized by irreversible cognitive decline and eventual patient death. Its primary pathological features include the formation of senile plaques in the cortex and neuronal loss, particularly in hippocampus (Zheng and Wang, 2025; Hardy, 2025). Despite extensive research, effective preventive and therapeutic measures for AD remain elusive, making it imperative to further investigate its underlying pathogenesis. Recent studies have linked abnormal expression of HtrA2 to the onset and development of progression AD. Immunohistochemical staining of brain tissue from control and AD patients revealed positive HtrA2 expression in the cerebral cortex and hippocampus of AD patients (Kawamoto et al., 2010). Double immunofluorescence analysis demonstrated significant co-localization of HtrA2 with both senile plaques and neurofibrillary tangles (Kawamoto et al., 2010). In addition to increased overall expression, the active form of HtrA2 was significantly elevated in AD brain tissue and positively correlated with acetylcholinesterase activity and the activity of acetylcholine-biosynthesizing enzymes (Darreh-Shori et al., 2019). These findings suggest a strong association between HtrA2 and the pathogenesis of AD. Amyloid beta (Aβ), the primary component of brain plaques in AD, plays a critical role in disease development, with its accumulation contributing to the pathophysiology of the disorder. Park et al. demonstrated an interaction between HtrA2 and Aβ through co-immunoprecipitation, identifying that the C-terminal region containing the PDZ domain of HtrA2 as the binding site for Aβ (Park et al., 2004). Additionally, HtrA2 was shown to effectively delay the aggregation of Aβ1-42 peptides, an effect independent of its protease activity (Kooistra et al., 2009). HtrA2 also directly cleaves amyloid precursor protein (APP) in mitochondria, preventing mitochondrial dysfunction caused by APP accumulation. Specifically, HtrA2 cleaves the C161 fragment of APP695 (amino acids 535–695) (Park et al., 2006). Collectively, these findings highlight HtrA2 as a key regulatory factor in the onset and development progression of AD.

### 5.3 Huntington’s disease (HTT)

HTT is a dominant autosomal neurodegenerative disorder caused by the expansion of CAG repeat sequences in the HTT gene. It is characterized by neurodegenerative changes, particularly in the striatum and cerebral cortex (Gulzar et al., 2025). As the number of CAG repeats increases, HTT becomes prone to misfolding, leading to the formation of insoluble aggregates, which are commonly found in the nucleus (forming neuronal nuclear inclusions, NIIs) and the cytoplasm of neurons after cell death (Kim et al., 2021). Emerging evidence suggests that HtrA2 may play a role in the pathogenesis of HTT. Reduced expression of HtrA2 has been observed in primary neurons expressing mutant HTT. Additionally, adenovirus-mediated overexpression of HtrA2 was shown to reverse mutant HTT-induced primary neuronal cell death (Inagaki et al., 2008). Goffredo et al. demonstrated that mutant Huntington protein triggers abnormal release of HtrA2 in brain-derived cells, leading to a decrease in cytoplasmic levels of IAP1 and XIAP. Furthermore, treatment with the HtrA2-specific inhibitor UCF-101 prevented IAP degradation and increased cell survival rate in Huntington cells (Goffredo et al., 2005). These findings suggest that inhibiting HtrA2-mediated cell death could represent a potential therapeutic strategy for HTT.

## 6 The agonists and inhibitors of HtrA2

Given the role of HtrA2 in apoptosis and disease progression, the development of specific inhibitors remains a key area of future research. Several compounds have been identified that inhibit the expression and activation of HtrA2, with UCF-101 being the most widely studied. UCF-101 is a highly selective inhibitor of HtrA2 serine protease activity, with a median inhibitory concentration of 9.5 μmol/L for HtrA2, and 200–500 μmol/L for other proteases (Bhuiyan and Fukunaga, 2008). UCF-101 can dose-dependently inhibit myocardial cell apoptosis at concentrations ranging from 0.6 to 1.8 μmol/kg, with the greatest protective effect observed at 1.5 μmol/kg. At this concentration, it has minimal impact on other proteases (Bhuiyan and Fukunaga, 2008), whereas at higher concentrations, its effects on cells are independent of inhibiting the HtrA2 pathway (Cilenti et al., 2003). Currently, UCF-101 is extensively used in animal and cell models to inhibit HtrA2 and improve disease progression in conditions such as heart disease (Bhuiyan and Fukunaga, 2007) and infectious diseases (Wang et al., 2018). In future studies using UCF-101 to investigate HtrA2 function, careful attention should be given to the concentration of UCF-101, and further validation through alternative methods, such as gene knockout or RNA interference, is recommended to confirm the inhibition of HtrA2 activity. In addition to UCF-101, several other compounds have been found to modulate HtrA2 expression. For instance, imatinib has been shown to upregulate HtrA2 expression at both the transcriptional and protein levels (Zhang et al., 2017), and imatinib mesylate may induce programmed cell death in BCR-ABL-positive human leukemia cells through HtrA2 (Okada et al., 2004). Furthermore, Li et al. demonstrated that deoxyarbutin interacts with HtrA2, inhibiting mitochondrial dysfunction via an HtrA2/PGC-1α-dependent pathway, thereby alleviating acute pancreatitis (Li et al., 2022). A summary of some of the agonists and inhibitors of HtrA2 is provided in Table 1.

**TABLE 1:** The agonists and inhibitors of HtrA2.

No.	Compounds	Effect on HtrA2	References
1	DL-3-n-butylphthalide	Inhibition	Huang et al. (2023)
2	Simvastatin	Inhibition	Yan et al. (2019)
3	Gastrodin	Inhibition	Nan et al. (2023)
4	Bortezomib	Inhibition	Arastu-Kapur et al. (2011)
5	Curcumin	Upregulation	Shankar and Srivastava (2007)
6	Isorhamnetin	Upregulation	Li et al. (2024)
7	Deoxyarbutin	Upregulation	Li et al. (2022)
8	Manganese	Upregulation	Jiang et al. (2014)
9	Imatinib	Upregulation	Zhang et al. (2017)

## 7 Conclusion

Recent advances in the study of HtrA2’s structure and function have significantly improved our understanding of its role regulating cell growth, apoptosis, mitochondrial homeostasis, and inflammation. Its abnormal function and expression are implicated in the onset and progression of neurodegenerative diseases. Future research should focus on identifying the substrate proteins of HtrA2. Additionally, while HtrA2 released into to the cytoplasm effectively activates the apoptotic pathway, its function within mitochondria requires further investigation. Moreover, the identification of specific agonists and inhibitors of HtrA2 remains a critical area of exploration. In conclusion, a deeper understanding of the structure and function of HtrA2 will provide valuable insights for targeting it in the treatment of neurodegenerative diseases, tumors, and other disorders.
